# A Sensitive, Highly Specific Novel Isothermal Amplification Method Based on Single-Nucleotide Polymorphism for the Rapid Detection of *Salmonella* Pullorum

**DOI:** 10.3389/fmicb.2020.560791

**Published:** 2020-10-07

**Authors:** Haiyan Shen, Junping Wen, Xinmeng Liao, Qijie Lin, Jianfeng Zhang, Kaifeng Chen, Shaojun Wang, Jianmin Zhang

**Affiliations:** ^1^Institute of Animal Health, Guangdong Academy of Agricultural Sciences, Guangzhou, China; Laboratory of Livestock Disease Prevention of Guangdong Province, Scientific Observation and Experiment Station of Veterinary Drugs and Diagnostic Techniques of Guangdong Province, Ministry of Agriculture, Guangdong, China; Maoming Branch, Guangdong Laboratory for Lingnan Modern Agriculture, Guangdong, China; ^2^National and Regional Joint Engineering Laboratory for Medicament of Zoonoses Prevention and Control; Key Laboratory of Zoonoses, Ministry of Agriculture; Key Laboratory of Zoonoses Prevention and Control of Guangdong Province; Key Laboratory of Animal Vaccine Development, Ministry of Agriculture; Guangdong Laboratory for Lingnan Modern Agriculture; College of Veterinary Medicine, South China Agricultural University, Guangzhou, China; ^3^School of Laboratory Medicine and Biotechnology, Southern Medical University, Guangzhou, China

**Keywords:** *Salmonella pullorum*, single-nucleotide polymorphism, rapid detection, RNase H2 enzyme, loop-primer probe introduced loop-mediated isothermal amplification

## Abstract

*S. Pullorum* (*Salmonella* enterica serovar Gallinarum biovars Pullorum) is an infectious pathogen that causes the acute systemic disease called Pullorum disease in poultry. This disease causes huge losses to the poultry industry and seriously affects the yield and quality of the chicken product. It is not easily distinguishable with fowl typhoid caused by *S.* Gallinarum (*Salmonella* enterica serovar Gallinarum biovars Gallinarum), hence the development of a specific and rapid detection method for this pathogen is highly desired. In this study, we propose a novel single-nucleotide polymorphism (SNP) detection strategy termed loop primer probe-introduced loop-mediated isothermal amplification (LP-LAMP) for *S.* Pullorum detection. Based on the original primer sets, we targeted the nucleotide position 237 of the rfbS gene sequence to design a new modified loop-primer probe with a ribonucleotide insertion, where activity of the enzyme ribonuclease H2 (RNase H2) is only activated when the probe is perfectly complementary, leading to the hydrolytic release of a quencher moiety and thus an amplified signal. The method exhibits robust specificity and a low detection limit as the copy number and genomic DNA is 21 copies/μL and 4.92 pg/μL, respectively. This method showed great performance in real sample testing of 130 samples of embryos, livers, and anal swabs from chickens in poultry farms. The experimental results are mainly consistent with traditional identification methods and a PCR method reported in the past. However, the other two methods still contain some false negative results, while our method is without miss detection. The entire closed-tube reaction process can be accomplished within 40 min at a constant temperature (61°C) without the need for expensive instruments or a complicated operation. The LP-LAMP strategy established in this study not only overcomes the existing difficulties of *S.* Pullorum rapid detection, it also provides a novel, sensitive, and highly specific detection platform for SNPs that is suitable for clinical use.

## Introduction

*S. Pullorum* (*Salmonella* enterica serovar Gallinarum biovars Pullorum) is a serious infectious pathogen that causes extensive economic loss to the poultry industry. Specifically, it causes the acute systemic condition Pullorum disease (PD) (Li et al., 2015), which affects the yield and quality of the chicken product. It is easily confused with fowl typhoid (FT) caused by *S.* Gallinarum (*Salmonella* enterica serovar Gallinarum biovars Gallinarum) as they are both septicemic diseases that affect the production of laying hens and have the same vertical and horizontal transmission characteristics (Barrow and Freitas Neto, 2011). Regrettably, traditional biological identification methods for *S.* Pullorum are time consuming and laborious (Ma et al., 2018). Furthermore, according to the serological typing rules of the White-Kauffmann-Le Minor scheme, *S.* Pullorum, *S.* Gallinarum, and *S.* Enteritidis (*Salmonella* enterica serovar Enteritidis) belong to serogroup D. Thus, they are difficult to distinguish by serological typing methods because of their similar antigenic formulae (Gast, 1997). Accordingly, the development of a highly specific rapid detection method for *S.* Pullorum is urgently needed.

At present, there are several polymerase-chain-reaction-based molecular biology methods for *S.* Pullorum detection, but these methods still cannot completely distinguish *S.* Pullorum from *S.* Gallinarum. Thus, in order to completely achieve specific detection of *S.* Pullorum, several researchers have exploited its single-nucleotide polymorphism (SNP) to provide a molecular target. In these studies, the mutation site of the rfbS gene has been regarded as an ideal molecular target to establish a method due to its great specificity (Park et al., 2002; Shah et al., 2005; Jeon et al., 2007; Ren et al., 2017). However, these established methods require a precise thermocycler which is expensive and must be operated by a trained professional. They also all need an open-tube to run in the agarose gel, which seriously hinders rapid detection. Therefore, establishing a sensitive and extremely accurate SNP detection method for the rapid detection of *S.* Pullorum is highly desired.

The commonly employed nucleic acid amplification method loop-mediated isothermal amplification (LAMP) exhibits superior and stable performance. This method can be used to amplify trace nucleic acids under constant temperature conditions in a short time (typically within 60 min). Furthermore, it provides an excellent detection performance without the need for advanced instrumentation and professional expertise (Mori et al., 2013; Youn et al., 2016; Mori and Notomi, 2020). However, the conventional LAMP method only amplifies gene fragments and cannot currently be used to detect SNPs (Srividya et al., 2019). Nevertheless, applying the LAMP method to the rapid detection of SNPs (Gill and Hadian Amree, 2020) would have a profound impact on pathogen detection, as well as far-reaching implications for genotyping, drug resistance testing, and clinical prognosis (Wang et al., 2020).

In this study, we developed a novel one-step and closed-tube LAMP method for the specific detection of SNP, termed loop primer probe-introduced loop-mediated isothermal amplification (LP-LAMP). We successfully validated our method by the specific detection of *S.* Pullorum. In doing so, we have demonstrated that the LP-LAMP method exhibits excellent sensitivity and is highly specific, making it potentially applicable to the detection of other SNPs.

## Materials and Methods

### Bacterial Strains

A total of 95 bacterial strains (Table 1) comprising different *Salmonella* serovars (55 strains of *S.* Pullorum, 6 strains of *S.* Gallinarum, 6 strains of *S.* Enteritidis, *S.* Corvallis, *S.* Derby, *S.* Rissen, *S.* London, *S.* Weltevreden, *S.* Typhimurium, *S.* Albany, *S.* Panama, *S.* Cerro, *S.* Kentucky, *S.* Havana, *S.* Mbandaka, *S.* Infantis, *S.* Goldcoast, *S.* Meleagridis, *S.* Agona, *S.* Indiana, *S.* Saintpaul, and *S.* Choleraesuis) and 9 non-*Salmonella* species (*Escherichia coli, Listeria monocytogenes, Staphylococcus aureus, Pseudomonas aeruginosa, Listeria iuanuii, Shigella, Riemerella anatipestifer, Campylobacter jejuni*, and *Campylobacter coli*) were used in a specificity test. Specific test strains were provided by the laboratory of the Veterinary College, South China Agricultural University, Guangzhou, China. Including the purchased standard strains or samples isolated from various markets in Guangzhou, all had been previously identified and stored at −40°C.

**TABLE 1:** *Salmonella* and non-*Salmonella* strains used in this study and specificity test results.

**Bacterial strain**	**Source**	**No.**	**Antigenic formulae**	**Specific test results**
*Salmonella* Pullorum	ATCC9120	1	1,9,12:-:-	+
	Laboratory stock	54		+
*Salmonella* Gallinarum	CICC21510	1	1,9,12:-:-	−
	Laboratory stock	5		−
*Salmonella* Enteritidis	ATCC13076	1	1,9,12:g.m:-	−
	Laboratory stock	5		−
*Salmonella* Corvallis	Laboratory stock	1	8,20:z4,z23: [z6]	−
*Salmonella* Derby	Laboratory stock	1	1,4,[5],12:f,g:[1,2]	−
*Salmonella* Rissen	Laboratory stock	1	6,7,14:f,g:-	−
*Salmonella* London	Laboratory stock	1	3,{10}{15}:l,v:1,6	−
*Salmonella* Weltevreden	Laboratory stock	1	3,{10}{15}:r:z6	−
*Salmonella* Typhimurium	ATCC 14028,	1	1,4,[5],12:i:1,2	−
*Salmonella* Albany	Laboratory stock	1	8,20:z4,z 24:–: [z45]	−
*Salmonella* Panama	Laboratory stock	1	1,9,12:l,v:1, 5:[R1…]	−
*Salmonella* Cerro	Laboratory stock	1	6,14,18:z4,z23:[1,5]:[z45],[z82]	−
*Salmonella* Kentucky	Laboratory stock	1	8,20:i:z6	−
*Salmonella* Havana	Laboratory stock	1	1,13,23: f,g,[s]:–:[z79]	−
*Salmonella* Mbandaka	Laboratory stock	1	6,7,14:z10:e,n,z15:[z37],[z45]	−
*Salmonella* Infantis	Laboratory stock	1	6,7,14:r:1,5:[R1…],[z37],[z45],[z49]	−
*Salmonella* Goldcoast	Laboratory stock	1	6,8:r:l,w	−
*Salmonella* Meleagridis	Laboratory stock	1	3,{10}{15}{15,34}:e,h:l,w	−
*Salmonella* Agona	Laboratory stock	1	1,4,[5],12:f,g,s:[1,2]:[z27],[z45]	−
*Salmonella* Indiana	Laboratory stock	1	1,4,12:z:1,7	−
*Salmonella* Saintpaul	Laboratory stock	1	1,4,[5],12:e,h:1,2	−
*Salmonella* Choleraesuis	Laboratory stock	1	6,7:c:1,5	−
*Escherichia coli*	Laboratory stock	1		−
*Listeria monocytogenes*	Laboratory stock	1		−
*Staphylococcus aureus*	CMCC26003	1		−
*Pseudomonas aeruginosa*	ATCC27853,	1		−
*Listeria iuanuii*	Laboratory stock	1		−
*Shigella*	Laboratory stock	1		−
*Riemerella anatipestifer*	Laboratory stock	1		−
*Campylobacter jejuni*	CICC22936	1		−
*Campylobacter coli*	CICC23925	1		−

*“+” means positive, “−” means negative.*

### Bacterial Culture and Genomic DNA Extraction

The frozen stocks of the bacterial strains were recovered on Xylose Lysine Tergitol-4 Agar (XLT-4 Agar) or Brain Heart Infusion (BHI) agar (Huankai Microbiology Technology Corporation, Guangdong, China) at 37°C overnight. The colonies were transferred to LB broth or BHI broth, and cultured at 37°C with constant shaking at a speed of 180 rpm for 16 h. *Salmonella* standard strains and non-*Salmonella* strains genomic DNA were extracted using a TIANamp Bacteria DNA Kit (TIANGEN Biochemical Technology Corporation, Beijing, China) and other *Salmonella* strains genomic DNA were extracted by the boiling method described previously (Youn et al., 2016), and then all samples were stored at −20°C until analysis.

### Construction of Standard Plasmids

PCR primers were designed according to the rfbS gene sequences of *S.* Pullorum (GenBank: LK931482.1) and *S.* Gallinarum (GenBank: AF442573) to amplify their full lengths. The T-vector was ligated with gene fragments at 16°C for 8 h after the fragment sequence identification, then transformed into DH5α cells. The plasmids were extracted using a commercial kit (Omega) according to the manufacturer’s instructions after sequencing verification. The plasmid concentrations were obtained by spectrophotometry and converted to copy numbers prior to storage at −20°C until use.

### Primer Design

**FIGURE 1:**
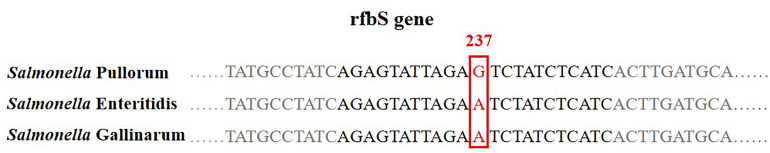
Single-nucleotide polymorphism at the 237th site of the rfbS gene for the *Salmonella* D serogroup.

LP-LAMP primers were designed with reference to the *S.* Pullorum rfbS gene sequence (GenBank: LK931482.1), and the rfbS gene position 237 was targeted (Figure 1) using Primer Explorer V5 (Table 2). All primers were synthesized by the Sangon Biotech (Shanghai) Co., Ltd. Their solutions (10 μM) were stored at −20°C until use.

**TABLE 2:** Primers sequences used in this study.

**Primer name**	**Sequence (5′–3′)**	**Length (bp)**
F3	AGGAACAATGAAGCTACCATA	21
B3	GGCAGTGATGTTCCACAAT	19
FIP	GTCTTCCATAGCAAGCAATAGTGTT-CACGACAGAAAATAATTGGATCG	48
BIP	ACCTGCAACAGCTTTAATAGAAAGC-GAATACTGCATCAAGTGATGAG	47
Loop primer	TCTTATGCCTATCAGAGTATTAGA	24
Loop primer probe	TCTTATGCCTATCAGAGTATT(FAM)AGAG(RNA base)TCTAT-Eclipse	30
ipaJ F	TACCTGTCTGCTGCCGTGA	19
ipaJ R	ACCCTGCAAACCTGAAATC	19

### Establishment and Optimization of the Basic Reaction System

The LP-LAMP reaction system was performed as follows: The system included 8U of Bst DNA polymerase, 100μmol of the MgSO_4_, 2.5μL of the buffer (10×) (New England Biolabs, Inc.), 0.1 U/μL of the RNase H2 Enzyme Kit (catalog:11-02-12-01, Integrated DNA Technologies), 20nmol of the dNTP (TransGen Biotech), and 2.5 μL of the DNA sample. All primers (10 μM) were synthesized by the Sangon Biotech (Shanghai) Co., Ltd. Deionized water was added until the mixture was 25 μL. The standard plasmids constructed for *S.* Pullorum and *S.* Gallinarum were used as detection objects, and the reaction procedure was performed at 1 cycle/min for a total of 60 cycles using a CFX96 Touch real-time PCR detection system (Bio-Rad). Signal acquisition was performed in FAM mode. We established a basic reaction system, and then optimized the dosage of loop primer, loop primer probe, and RNase H2 enzyme independently.

### Detecting the Limit of the LP-LAMP Method

In order to define the detection limit of the optimized LP-LAMP method, positive plasmid solutions with concentrations ranging from 2.1 × 10^6^ to 2.1 copies/μL were prepared by a 10-fold serial dilution in deionized water. Meanwhile, the genomic DNA of the *S.* Pullorum strain (ATCC9120) was 10-fold serially diluted from 49.2 ng/μL to 0.492 fg/μL in deionized water. All samples above were analyzed in triplicate and the results were used to establish a respective standard curve.

### Specificity of the LP-LAMP Method

Genomic DNA extracted from 55 strains of *S.* Pullorum, 40 strains of various *Salmonella* serovars, and other different non-*Salmonella* pathogens (Table 1) were used to evaluate the inclusivity or specificity of the LP-LAMP method.

### Application of the LP-LAMP Method

A total of 130 real samples, which included 70 embryos, 30 livers, and 30 anal swabs, were collected from poultry from a commercial chicken farm in Guangzhou, China according to the Chinese national standard (GB4789.4-2016). All samples were cultured in buffered peptone water (BPW) for pre-enrichment at 37°C for 8 h. The bacterial culture was subsequently inoculated into a Selenite Cystine Broth (SC) and cultured at 37°C for 18 h. Then the bacteria broth was streaked on a *Salmonella Shigella* agar and incubated continuously at 37°C for 14 h. Then using the *Salmonella* biochemical identification kit we performed serotyping rapid agglutination (The above reagents were purchased from the Huankai Microbiology Technology Corporation, Guangdong, China). After the samples were pre-enriched by the BPW overnight they were extracted by the boiling method and used as reaction templates to evaluate the actual application performance of the LP-LAMP method on natural-occurring *Salmonella* isolates. Simultaneously, according to the method reported by Xu et al. (2018), PCR primers targeting the *S.* Pullorum ipaj gene were synthesized (Sangon Biotech) to detect the same samples (Table 2). We then compared the detection results of the traditional identification method, the PCR method, and the LP-LAMP method.

## Results

### Principle of the LP-LAMP Method and Establishment of the Basic Reaction System

**FIGURE 2:**
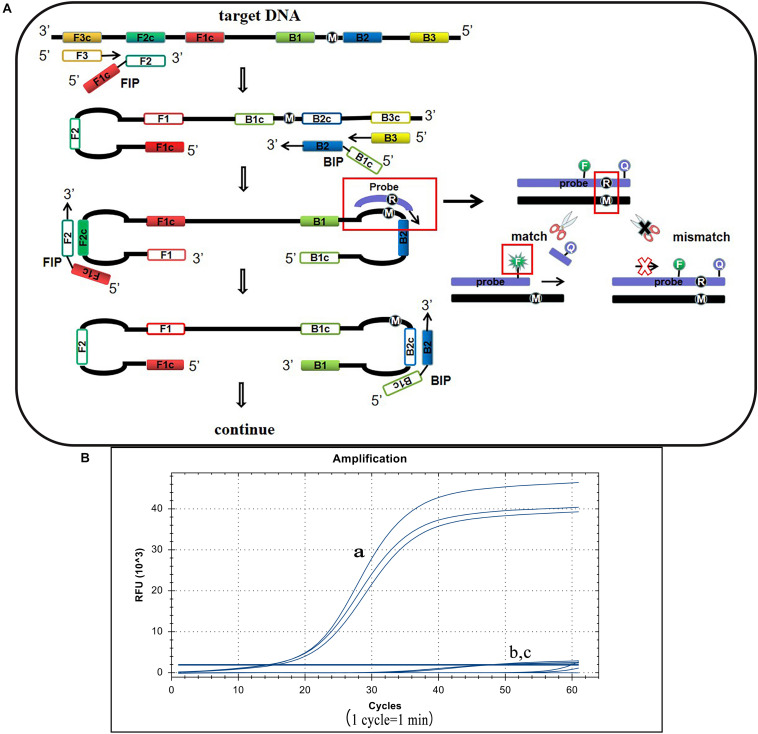
Principle of the LP-LAMP method and the basic reaction system. **(A)** The principle of the novel SNP detection method (LP-LAMP) established in this study. **(B)** Basic reaction system. (a) *Salmonella* Pullorum standard plasmid. (b) *Salmonella* Gallinarum standard plasmid. (c) Negative control.

*S.* Pullorum, *S.* Gallinarum, and *S.* Enteritidis belong to the *Salmonella* D serogroup, where the rfbS gene is a specific sequence in this serogroup. The 237th position of the *S.* Pullorum rfbS gene is a guanine while in *S.* Gallinarum and *S.* Enteritidis the gene is an adenine. Other *Salmonella* serotypes or non-*Salmonella* pathogens do not contain this gene or vary widely (Figure 1). Hence we targeted this site of *S.* Pullorum to design a new reporter dye and quencher-modified allelic discrimination loop primer probe with a ribonucleotide insertion based on the original LAMP primer sets. When the ribonucleotide perfectly matched with the mutant site, the hydrolytic mechanism of the RNase H2 enzyme was activated, releasing the quencher to provide an amplified signal. Conversely, no signal was generated for a mismatching ribonucleotide (Figure 2A). Thus, robust specific detection of SNP was achieved. We successfully validated our principle by establishing a basic reaction system using standard plasmids (Figure 2B), where only the *S.* Pullorum standard plasmids were successfully detected while the *S.* Gallinarum standard plasmid failed to generate a curve. This result proves that the mutant template can effectively detect plasmids while the wild-type template cannot cause interference by this novel SNP detection method.

### Optimization Results of Basic Reaction System

**FIGURE 3:**
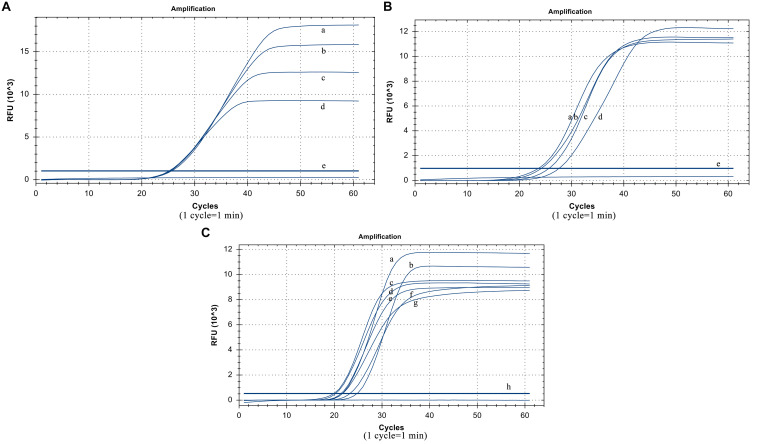
Optimized results. **(A)** Usage of the loop primer probe. (a) 20 pmol, (b) 15 pmol, (c) 10 pmol, (d) 5 poml, (e) Negative control. **(B)** Usage of the loop primer. (a) 15 pmol, (b) 10 pmol, (c) 20 pmol, (d) 5 pmol, (e) Negative control. **(C)** Usage of Rnase H2 enzyme. (a)0.06 U/μL, (b) 0.02 U/μL, (c) 0.12 U/μL, (d) 0.1 U/μL, (e) 0.14 U/μL, (f) 0.04 U/μL, (g) 0.08 U/μL (h) Negative control.

The suitable collocation of the loop primer and the loop primer probe is crucial to reinforce reaction efficiency. When the same dilution template was used and the other conditions were unchanged, the cycle thresholds of different loop primer probe dosages were very similar. Considering the actual cost of use, the optimized dosage of the loop primer probe was 5 pmol (Figure 3A). When the amount of the loop primer probe used was 5 pmol, the best performing dosage of the loop primer was 15 pmol (Figure 3B), which means their optimal ratio is 1:3.

The RNase H2 enzyme dosage optimization results show that it has the smallest cycle threshold at 0.06 U/μL, and the gap was not particularly large compared to those at other dosages. Thus, from the perspective of overall performance and cost reduction, we selected 0.06 U/μL as the optimal reaction volume (Figure 3C). The reaction temperature was 61°C.

### Detection Limit Test Results

**FIGURE 4:**
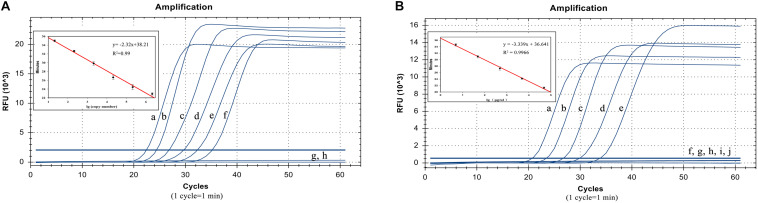
Detection limit test results. **(A)** LP-LAMP method for the detection of plasmid concentrations. Diluted *Salmonella* Pullorum plasmid was used at the following concentrations: (a) 2.1 × 10^6^ copies/μL, (b) 2.1 × 10^5^ copies/μL, (c) 2.1 × 10^4^ copies/μL, (d) 2100 copies/μL, (e) 210 copies/μL, (f) 21 copies/μL, (g) 2.1 copies/μL. (h) Negative control. **(B)** LP-LAMP method for the detection of genomic DNA, diluted *Salmonella* Pullorum genomic DNA was used at the following concentrations: (a) 49.2 ng/μL, (b) 4.92 ng/μL, (c) 492 pg/μL, (d) 49.2 pg/μL, (e) 4.92 pg/μL, (f) 492 fg/μL, (g) 49.2 fg/μL, (h) 4.92 fg/μL, (i) 0.49 2fg/μL, (j) Negative control.

The plasmid concentrations from 21 copies/μL to 2.1 × 10^6^ copies/μL were successfully amplified. Thus, the detection limit was 21 copies/μL and analysis can be completed within 40 min. The standard curve equation was *y* = −2.32*x* + 38.21 (*R*^2^ = 0.99). Thus, there was an excellent correlation between the copy number and reaction time (Figure 4A).

In addition, the sensitivity was also evaluated by detecting the serially diluted genomic DNA from *S.* Pullorum (ATCC 9120). The results showed that concentrations from 4.92 pg/μL to 49.2 ng/μL were successfully detected. This revealed that at least 4.92 pg/μL of the bacterial genomic DNA was required to identify and distinguish *S.* Pullorum using the LP-LAMP method. The standard curve equation was *y* = −3.339x + 36.641 (*R*^2^ = 0.99). Thus, there was a great correlation between genomic DNA concentrations and reaction time as well (Figure 4B). The equations above can be used for accurate quantification of unknown samples.

### Specificity Test Results

**FIGURE 5:**
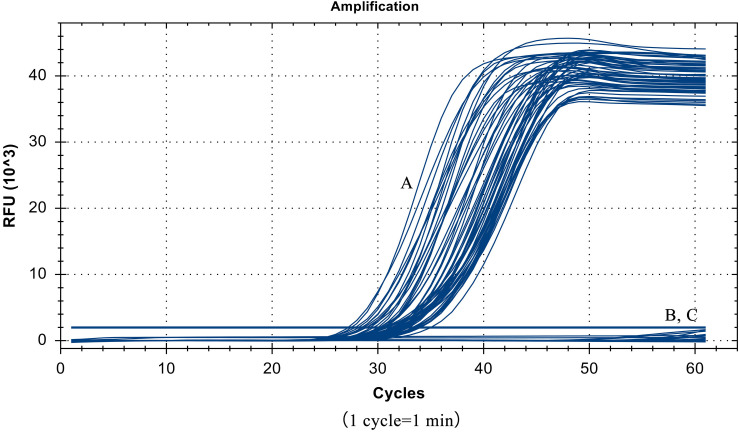
Specificity test results, (A) 55 strains of *Salmonella* Pullorum. (B) 40 strains of other serotypes of *Salmonella* and non-*Salmonella* species. (C) Negative control.

Despite the similarity of the *S.* Pullorum target gene sequence to those of *S.* Gallinarum and *S.* Enteritidis, only a total of 55 strains of *S.* Pullorum generated the specific amplification curve. In addition, the other 40 interfering strains of different *Salmonella* serovars and non-*Salmonella* strains were not detected (Table 1 and Figure 5). These results suggest that the developed LP-LAMP method had an excellent 100% inclusivity and specificity.

### Application Results of the LP-LAMP Method

To evaluate the application of the LP-LAMP method, the 130 isolates of unknown serovars samples were collected from embryo, liver, and anal swabs of poultry in naturally contaminated chicken farms to use as a detect template. Results show that 42 samples (33 embryos, 7 livers, and 2 anal swabs) were detected as positive by all three methods while 58 samples (25 embryos, 10 livers, 23 anal swabs) were all negative. A total of 21 samples (9 embryos, 8 livers, 4 anal swabs) were detected as positive by the two molecular biological methods but were not detected by traditional identification methods. A total of 4 samples (2 embryos and 2 livers) were detected as positive by the traditional identification method and the LP-LAMP method established in this paper, but the PCR method had no bands. In addition, there were 5 samples (1 embryo, 3 livers, and 1 anal swab) that were only detected by the LP-LAMP method while the two other methods missed detection (Table 3).

**TABLE 3:** Comparison of the results of testing real samples using different methods.

**Sources (amounts)**	**Sample amounts (130)**	**Traditional identification methods**	**PCR method**	**LP-LAMP method**
	33	+	+	+
	25	−	−	−
Embryo (70)	9	−	+	+
	2	+	−	+
	1	−	−	+
	7	+	+	+
	10	−	−	−
Liver (30)	8	−	+	+
	2	+	−	+
	3	−	−	+
	2	+	+	+
	23	−	−	−
Anal swab (30)	4	−	+	+
	0	+	−	+
	1	−	−	+
Reference		GB4789.4-2016	Xu et al., 2018	This study

*+, Salmonella Pullorum positive.* −, *Salmonella Pullorum negative.*

## Discussion

PD has a huge impact on the development of the poultry industry. It has been largely eradicated from many countries of Europe and North America. However, it continues to occur and cause great economic loss to the poultry industries of many developing countries, including China (Shah et al., 2005), where frequent outbreaks of PD still affect many commercial poultry farms (Hu et al., 2019) and seriously affect the production of chicken products. Therefore, methods for the early detection of *S.* Pullorum are required. In this study, we have successfully overcome the long-standing *S.* Pullorum detection bottlenecks and used our novel strategy to address the fact that conventional LAMP cannot be used for single nucleotide mutation site detection.

The conventional LAMP method is a nucleic acid amplification test that is extensively applied in the rapid detection of bacterial, fungal, parasitic, and viral agents (Yang et al., 2018). In this method, primers for six regions of the target gene were designed and can complete the amplification process in a short time. However, it can only achieve the amplification of large segments of a gene and is unable to detect single nucleotide mutations. Herein, we successfully solved this problem by designing a loop primer probe with a ribonucleotide insertion, which is cleaved by RNase H2 enzyme only when the base sequence perfectly matches that of the mutant target. Through this design, the specificity of the probe is greatly enhanced, allowing the SNP to be unambiguously identified. This may expand the usage scenarios of the LAMP method for genotyping, drug resistance gene mutation detection, early accurate diagnosis, and clinical prognosis of many human and animal diseases.

We had successfully applied our novel strategy to solve the long-standing difficulties of *S.* Pullorum detection through the specific detection of the rfbS gene, which has been identified as a robust genetic marker for the identification of *S.* Pullorum based on its polymorphisms. Among *Salmonella* serogroups A, B, C2, and D, the rfbS genes encoding paratose synthase have been reported to be used to detect the *Salmonella* serogroup D. According to previous research and based on our sequence analysis, there were regular changes in the rfbS gene in the *Salmonella* serogroup D strains. Specifically, at position 237, all *S.* Pullorum isolates had a guanine, while the other *Salmonella* serogroup D strains (including *S.* Gallinarum and *S.* Enteritidis) had an adenine in this position. Other Salmonella serotypes or other pathogens have a dissimilar sequence or even do not contain this gene. Hence, the polymorphisms at positions 237 represent a potential robust molecular target for the specific detection of *S.* Pullorum (Luk et al., 1993; Shah et al., 2005; Ren et al., 2017). Furthermore, this target is present in all *S.* Pullorum strains, unlike the gene ipaj (Xu et al., 2018) which only exists in a plasmid not carried by all the strains, or the ratA gene which is not specific enough to be found in other *Salmonella* serotypes genomes, our method will not overlook any strains or cause false detection (Batista et al., 2013, 2016). Through the choice of this ideal detection target and the original design reaction scheme, our method has great inclusivity due to the fact that all 55 strains of *S.* Pullorum were successfully detected while 31 strains of other different *Salmonella* serovars and 9 strains of non-*Salmonella* pathogens did not cause interference, even when there was only one base difference in the target gene, like those for *S.* Gallinarum and *S.* Enteritidis. Thus, unlike the related method established by Xiong et al. (2016, 2017), which cannot distinguish *S.* Pullorum from *S.* Gallinarum, we have successfully realized completely specific *S.* Pullorum detection.

Furthermore, our experimental results show that our LP-LAMP method makes perfect use of the sensitivity of the basic LAMP strategy, only adding a new fluorophore-modified probe while retaining the original LAMP reaction primer sets and matching the dosage of the loop primer and loop primer probe. The minimum detectable copy number is 21 copies/μL, which is comparable with the sensitivity of the PCR-HRM method reported by Ren et al. (2017) (34 copies/μL) and 100 times more sensitive than the PCR method. In addition, the detection limit for genomic DNA reaches 4.92 pg/μL, which is better than some previous reports (Yang et al., 2018). Therefore, our method will not miss the detection of real samples. The test results of 130 real samples from embryos, livers, and anal swabs show that our method conforms to traditional identification methods and the PCR method (Xu et al., 2018). Among them, 42 samples were detected positive by all three methods while 58 samples were all negative. Moreover, our method successfully avoids the problem of missing detection because of insufficient sensitivity like traditional identification methods and the PCR method. Results show that a total of 21 samples can only be detected as positive by two molecular biological methods but are not detected by the traditional identification method, while 4 samples were only detected as positive by the traditional identification method and the LP-LAMP method established in this paper, while the PCR method had no bands. In addition, there are 5 samples can only be detected by the LP-LAMP method while the two other methods provided a false negative. Compared with the traditional identification method and the reported molecular biology PCR method, we have a higher sensitivity which can avoid missed detection in actual use. Simultaneously, with our method, the superior sensitivity detection for *S.* Pullorum has prospects that can be achieved without the thermocycler that other existing reports required. The whole reaction process can be completed with cheaper constant temperature instruments to maintain a constant reaction temperature (61°C) instead of a thermocycler, which can greatly reduce the cost of the method for clinical use.

In addition to exhibiting excellent sensitivity, the entire detection process for the LP-LAMP method can be completed in only 40 min, much shorter than traditional identification methods that require several days to yield results (Proux et al., 2002). Furthermore, compared with previously reported *S.* Pullorum detection methods based on SNP, such as PCR-RFLP (Park et al., 2002) and allele-specific PCR (Shah et al., 2005), which require complex and time-consuming follow-up operation processes, our method eliminates the problem of open-tube for electrophoresis. The whole detection process can be completed under closed-tube conditions after a one-step sample addition process. Thus, our succinct operation process meets the needs of PD treatment and clinical study, as well as presenting the prospect of application to other SNPs.

## Conclusion

In this study, we have successfully applied the LP-LAMP method for SNP detection and verified our novel strategy by detecting *S.* Pullorum. The advanced sensitive and specific LP-LAMP method realizes rapid SNP detection and solves previous *S.* Pullorum detection problems. It is highly suitable for primary-level use in commercial chicken farms. In addition, this method shows promise for applications in other types of single nucleotide mutant detection, which has broad application prospects in genotyping, drug resistance gene mutation detection, early accurate diagnosis, and clinical prognosis of many human and animal diseases.

## Data Availability Statement

The raw data supporting the conclusions of this article will be made available by the authors, without undue reservation.

## Ethics Statement

Our protocol was used to detect *Salmonella* Pullorum identified from dead poultry samples, which does not include ethical issues related to live animals. The samples collection process complies with relevant national standards and does not involve animal experiments.

## Author Contributions

HS, JW, and JMZ conceived and designed the experiment. HS and JW carried out the experiment and performed the analysis. HS, JW, XL, QL, JFZ, KC, and SW contributed to sample preparation and collected the data. JW wrote the manuscript. HS, JW, and JMZ revised the manuscript. JMZ funded the project. All authors discussed the results and commented on the manuscript.

## Conflict of Interest

The authors declare that the research was conducted in the absence of any commercial or financial relationships that could be construed as a potential conflict of interest.
